# Decoding the Gut Microbiome in Companion Animals: Impacts and Innovations

**DOI:** 10.3390/microorganisms12091831

**Published:** 2024-09-04

**Authors:** Harsh Shah, Mithil Trivedi, Tejas Gurjar, Dipak Kumar Sahoo, Albert E. Jergens, Virendra Kumar Yadav, Ashish Patel, Parth Pandya

**Affiliations:** 1 TREE Lab, Department of Biomedical and Life Sciences, School of Science, Navrachana University, Vadodara 391410, Indiamithilt9727@gmail.com (M.T.);; 2 Department of Veterinary Clinical Sciences, College of Veterinary Medicine, Iowa State University, Ames, IA 50011, USA; ajergens@iastate.edu; 3 Marwadi University Research Center, Department of Microbiology, Faculty of Sciences, Marwadi University, Rajkot 360003, India; yadava94@gmail.com; 4 Department of Life Sciences, Hemchandracharya North Gujarat University, Patan 384265, India; uni.ashish@gmail.com

**Keywords:** gut microbe, companion animal, disease, health, factors

## Abstract

The changing notion of “companion animals” and their increasing global status as family members underscores the dynamic interaction between gut microbiota and host health. This review provides a comprehensive understanding of the intricate microbial ecology within companion animals required to maintain overall health and prevent disease. Exploration of specific diseases and syndromes linked to gut microbiome alterations (dysbiosis), such as inflammatory bowel disease, obesity, and neurological conditions like epilepsy, are highlighted. In addition, this review provides an analysis of the various factors that impact the abundance of the gut microbiome like age, breed, habitual diet, and microbe-targeted interventions, such as probiotics. Detection methods including PCR-based algorithms, fluorescence in situ hybridisation, and 16S rRNA gene sequencing are reviewed, along with their limitations and the need for future advancements. Prospects for longitudinal investigations, functional dynamics exploration, and accurate identification of microbial signatures associated with specific health problems offer promising directions for future research. In summary, it is an attempt to provide a deeper insight into the orchestration of multiple microbial species shaping the health of companion animals and possible species-specific differences.

## 1. Introduction

Domesticated animals are the result of generations of selective breeding and genetic adaptation to coexist with humans [1]. These companion animals permanently reside in human communities and provide companionship, entertainment, work, and psychological support [2,3]. Pet ownership is increasing globally, with a growing tendency to view pets as family members. Notably, around 90% of pet owners view their companion animals as integral and fully functional members of their families [4]. An overall survey of the world population review has shown an understanding of the preference for companion animals as pets in general (Supplementary Figure S1) and dogs and cats (Supplementary Figure S2).

The digestive system of animals and humans harbors numerous bacteria as well as viruses, fungi, and other microorganisms (e.g., protozoa and algae). Importantly, the structure and function of the gut microbiome are strongly influenced by different dietary components (carbohydrates, fats, proteins, minerals, vitamins, etc.), food additives, cooking, and processing, and these changes are closely related to maintaining the health of the host [5]. Consequently, the gut serves as a stable ecological niche for these inhabiting bacteria, relying on the host’s physiological processes, such as feeding and reproduction, for their basic biological functions [6,7,8,9]. The diverse microorganisms, including bacteria, protozoa, bacteriophages, fungi, archaea, and eukaryotic viruses, which inhabit and colonise the anatomical structures of humans and other animals, constitute more than a mere collection of microbes. The microbiome encompasses the entirety of microorganisms, their genes, and their metabolites [10]. Day-by-day research focuses on understanding the role of gut microbiota in shaping the host’s health. Many association studies have been carried out on human health, which include metabolic disorders like diabetes [11,12], obesity [13,14], kidney disease [15,16], atopic disorders [17], chronic enteropathy (CE) [18,19], immune-mediated disorders [20,21], and allergic reactions [22]. Additionally, the species diversity and composition of the gut microbiome are affected by many factors like diet [23], antibiotics [24], probiotics [25], age of the animal [26], physical activity [27], gender, and breed [28]. There are also differences in the mucosal versus luminal abundance of bacteria. These factors are detailed in the further sections.

The gut microbiome is dominated by strict or facultative anaerobic bacteria, particularly in the large intestine [29]. Firmicutes (low-G + C Gram-positive bacteria), Fusobacteria (Gram-negative anaerobic bacilli), and Bacteroidetes (Gram-negative rod-shaped) are the most abundant phyla in dogs and cats (Table 1).

The gut microbiota produces two primary metabolites: short-chain fatty acids (SCFAs) and trimethylamine-*N*-oxide (TMAO) [29,31]. The genus *Fusobacterium* is associated with good health in dogs [32], while it has been linked as one of the causative agents leading to colorectal cancer [33] and IBD [34] in humans. Fusobacteria is considered to have a crucial role in the gut metabolism of carnivorous animals [35] because of their unique ability to break down protein and amino acids to generate branched-chain volatile fatty acids, SCFAs [36]. In the gastrointestinal (GI) tract of dogs, *Fusobacterium* species, such as *F. perfoetens* and *F. mortiferum*, are abundantly present [37] (Table 2), while *Fusobacterium* makes up approximately 20% of the overall relative abundance [31]. The gut microbiota significantly impacts various facets of animal health, encompassing innate immunity, appetite regulation, and energy metabolism [38,39,40,41,42,43,44]. The strategic manipulation of the gut microbiome through interventions such as probiotics or dietary fibre holds promise for enhancing overall health and potentially mitigating the prevalence of obesity [40].

This review focuses on studies investigating the impact of the gut microbiome on health outcomes in companion animals, primarily in dogs and cats. It emphasises research on pathological conditions such as inflammatory bowel disease, obesity, and neurological disorders, encompassing a range of ages, diets, and probiotic interventions. To maintain a rigorous and relevant analysis, studies involving non-companion species, animals with severe systemic diseases, impact of antibiotic use, or mixed/uncontrolled diets were excluded. Additionally, we omit research published before 1990, non-English language studies, and those with overlapping experimental treatments, ensuring a refined and focused scope.

## 2. Techniques Used for the Detection of Gut Microbiome Abundance

Using a recently developed polymerase chain reaction (PCR)-based algorithm called the “Dysbiosis Index,” veterinarians may quantify the magnitude of microbial imbalance (dysbiosis) in faeces and monitor the progression of microbial imbalances in response to treatment in dogs and cats [51]. Alterations or perturbations in the composition of the microbiota have a discernible impact on the functionality of the immune system. Consequently, the manipulation or modulation of the gut microbiome holds potential therapeutic value in the management of GI disorders [52].

Fluorescence in situ hybridisation (FISH) is a widely employed method that utilises fluorescent dye-labelled oligonucleotide probes that hybridise to bacterial ribosomal RNA within targeted bacterial groups. This technique permits the identification, quantification, and spatial distribution of microbes in tissue [44,53,54]. The principal advantage of FISH is the ability to spatial visualise bacterial localisation.

Next is 16S rRNA gene sequencing: DNA shotgun sequencing (metagenomics) and other techniques are included in next-generation sequencing (NGS). Only a small number of studies employed deep DNA shotgun sequencing, while most studies evaluating the gut microbiota in companion animals used 16S rRNA gene sequencing. To be sure, 16S rRNA gene sequencing is the most widely used sequencing method for evaluating gut bacteria in dogs and cats. In a nutshell, intestinal materials, including biopsies, luminal content, or faecal samples, are used to extract DNA. The conserved sections on either side of the many variable regions that make up the 16S rRNA gene. The variable region between these conserved sections is amplified by using bacterial primers. Theoretically, it is possible to amplify DNA from both known and unknown bacteria in the sample and subsequently sequence the variable regions by specifically targeting the conserved regions. The NGS technologies have helped us to characterise bacterial species and their interactions between the host and gut microbiome. Using this technique, the microbiota of companion animals (dogs and cats) has been described, including those of the gastrointestinal tract (GIT) [31,55,56,57], skin [58], oral cavity [59,60], nasal cavity [61], and vagina [62].

The gut microbiome can also be defined by bacterial culture in some instances. However, because only conventional bacterial medium and/or restricted anaerobic procedures are utilised in traditional bacterial culture, the number of intestinal bacteria is greatly underestimated when performed in veterinary diagnostic laboratories. Commercial diagnostic laboratories have reported the isolation of just a tiny percentage of bacterial species from the faeces of clinical patients. Unfortunately, physicians frequently mistakenly perceive these bacteria as pathogens since they have been identified from samples of clinical patients.

The existing methodologies for detection exhibit inherent limitations that necessitate their transcendence, while a comprehensive understanding of the underlying mechanism is imperative.

## 3. Factors Influencing the Gut Microbiome

**Figure 1:**
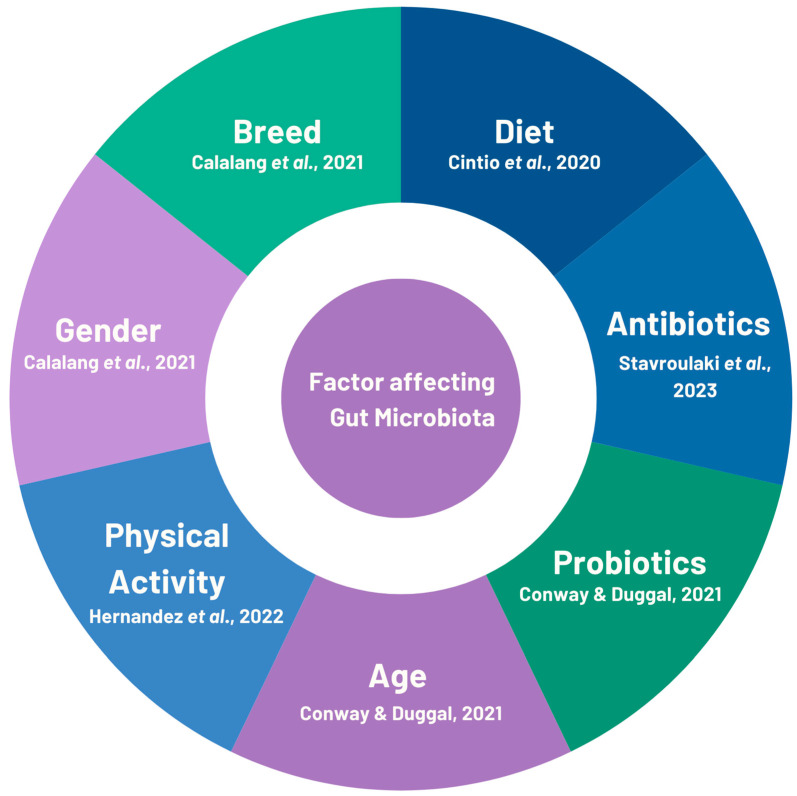
Accountability of different factors affecting the gut microbiota in canines and felines. The information displayed in the figure was derived from previously published studies [23,24,26,27,28].

The composition of the gut microbiota can be substantially influenced by a variety of factors, such as age, diet, and the use of probiotics and medications, particularly antibiotics, as demonstrated by various animal models (Figure 1).

### 3.1. Age and Breed

**Figure 2:**
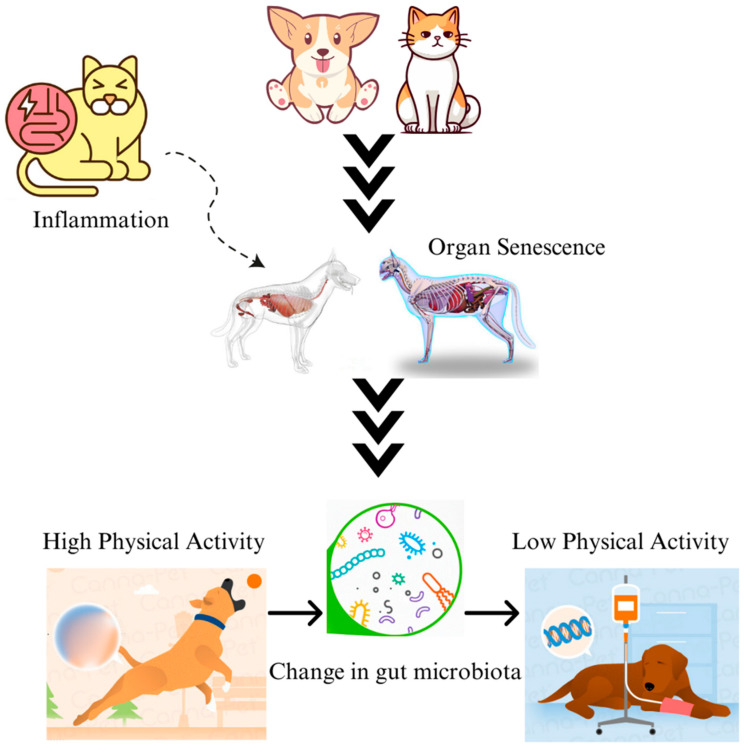
Companion animals may become less active due to gastrointestinal inflammation triggered by age- or diet-induced dysbiosis. Intestinal damage brought on by mucosal inflammation due to the changes in the gut microbiome might also result in decreased physical activity.

The influence of age, whether in humans or companion animals, emerges as a prominent determinant of the notable alterations observed in the composition of gut microbial communities, suggesting a discernible decline in microbial diversity [63]. The phenomenon of organ senescence, characterised by increased inflammatory reactions, engenders a persistent alteration in the composition and functionality of the gut microbiome, thereby exerting a discernible impact on the physical activity and pharmacological intake patterns of the organism in question (Figure 2) [64]. In dogs, after weaning, the presence of Fusobacteria becomes more prevalent, with *Fusobacterium perfoetens* being approximately twice as abundant in the 6–10-year-old group compared to the 0.5–1-year-old group (14.3% vs. 7.2%). While such an increase in abundance is positively associated with the age of the dogs [64,65], there is also a potential association between the increase in levels of Fusobacteria and the use of meat-based foods after the weaning stage in dogs [35,64].

Similarly, in cats, between weeks 18 and 42, there were notable shifts in the average abundance of the four most common genera: *Bifidobacterium*, *Lactobacillus*, *Prevotella*, and *Bacteroides* [66,67]. The levels of *Bacteroides* and *Prevotella* showed a significant increase with age, while the levels of *Bifidobacterium* and *Lactobacillus* showed a significant decrease. By the time kittens reached 18 weeks of age, the microbiome was primarily composed of *Lactobacillus* (35%) and *Bifidobacterium* (20%). The increased presence of *Lactobacillus* and *Bifidobacterium* during the earlier time points (8–17 weeks) can be attributed to the impact of the milk-feeding/weaning period. By the time they reached 42 weeks of age, *Bacteroides* accounted for 16% of the population, followed by *Prevotella* at 14% and *Megasphaera* at 8.4%. The abundance of *Megasphaera* experienced a significant increase over time, rising from 0.1% and 0.2% at week 18 and week 30, respectively, to 8.4% at week 42 [66,67]. Studies have also demonstrated breed-specific differences in the canine intestinal microbiome. For example, Hooda et al. [68] compared three breeds: Maltese, Poodle, and Miniature Schnauzer, where the richness and abundance of *Fusobacterium* differed and the abundance of Firmicutes was lowest in Maltese dogs. On the contrary, Li et al. [69] showed the breed-specific differences using 16S rRNA gene sequencing techniques in the gut microbiota of Felinae and Ragdoll cats and proved that beneficial microbes like *Enterococcus*, *Lactobacillus*, *Streptococcus*, *Roseburia*, and *Blautia*, was significantly abundant in the Ragdoll group than in the Felinae group, suggesting its use in specific designing of probiotic.

### 3.2. Gender

There has been a significant increase in research indicating variations in gut microbiota between males and females, as observed in both animals (mostly in murine models) and humans [70,71]. For instance, a study conducted on non-obese diabetic (NOD)/ShiLtJ mice found that certain bacterial families such as *Kineosporiaceae*, *Peptococcaceae*, *Porphyromonadaceae*, *Veillonellaceae*, *Lactobacillaceae*, *Peptostreptococcaceae*, *Bacteroidaceae*, *Cytophagaceae*, and *Enterobacteriaceae* were more abundant in male mice compared to female mice [70]. Microbial dysbiosis caused by Bilateral ovariectomy has been reported in murine models [72,73], while in humans, bilateral ovariectomy has been linked to a higher presence of *Clostridium bolteae* [71]. There was a direct correlation between the level of non-ovarian systemic estrogens and the abundance of faecal Clostridia, which included non-*Clostridiales* and three genera in the *Ruminococcaceae* family [74]. In companion animal studies, it was found that the relative abundance (RA) of *Firmicutes* showed similarities between spayed females and castrated males, which was significantly higher in these groups compared to normal female and male dogs. In addition, there was a higher proportion of *Firmicutes* in male dogs compared to females in terms of their RA. On the other hand, male dogs had a lower RA of Bacteroidetes compared to females, but it was higher than that of castrated dogs (spayed females and castrated males). There was a higher RA of *Fusobacterium* in normal dogs (both female and male) compared to castrated dogs (spayed females and castrated males) [75].

### 3.3. Physical Activity

Research has shown a strong connection between physical activity and improved metabolic health. Human studies have indicated that physical activity can lead to a higher presence of beneficial gut microbes, such as Clostridiales [76]. Research has revealed a positive correlation between regular exercise and a higher presence of bacteria in the Erysipelotrichaceae family. Engaging in active transportation was found to have a positive impact on the levels of *Phascolarctobacterium*, while simultaneously reducing the levels of *Clostridium*. Engaging in active transportation for longer durations was found to be linked to a reduction in the presence of the Clostridiaceae family [76]. Several clinical trials have investigated the effects of exercise and dietary intervention on the canine gut microbiota [77,78]. The study by Kieler et al. did not find any evidence of exercise impacting the gut microbiota composition in a weight-loss programme that utilised a commercial high-protein, low-fat, and high-fibre dry diet [77]. In contrast, especially during exercise-related activity, the addition of glucosamine affects the composition of the microbiome in sled dogs [79]. When examining the taxonomic composition at the family level, it becomes evident that sled dogs who consume glucosamine experience a reduction in *Lactobacillaceae* and *Anaerovoracaceae*. In glucosamine-supplemented dogs, the abundance of *Sellimonas*, *Eubacterium brachy*, and *Parvibacter* was found to be reduced, especially after activity [79].

### 3.4. Antibiotics

Even though antibiotics were originally designed to combat specific pathogens in animals and humans, their molecular targets, such as the ribosome, RNA polymerase, and also cell wall, are highly conserved among different bacterial species. As a result, the use of antibiotics can affect both harmful and harmless bacteria, leading to disturbances in the ecological balance that plays a crucial role in various metabolic processes. Tackling bacterial infections has become more difficult, as different strains of bacteria are showing a growing resistance to antimicrobial treatments [80,81]. It is worth mentioning that the disruption of the gut community caused by antibiotic treatment allows the proliferation of harmful pathogens that can cause gastrointestinal diseases, including vancomycin-resistant *Enterococcus*, *Salmonella* spp., and drug-resistant Enterobacteriaceae, *Clostridioides difficile* [82].

Research conducted on both humans and dogs consistently shows that metabolic transformations due to antibiotic treatment are linked to noticeable reductions in the presence of key members from the Bacteroidetes, Firmicutes, and Actinobacteria, particularly *Faecalibacterium* [27,83,84], as they play a vital role in the gut microbiome’s metabolic processes. Studies on antibiotic treatment in murine models and humans showed that it often leads to a decline in their ability to ferment carbohydrates (leading to decreased SCFA production) and transform bile acids (resulting in more primary bile acids production) [85,86,87]. Using advanced-omic or quantitative polymerase chain reaction (q-PCR)-based techniques to investigate the effects of tylosin, metronidazole, or metronidazole in combination with enrofloxacin revealed a notable increase in the dysbiosis index and a lasting disturbance in bacterial abundances and a decrease in species diversity, even after discontinuation of the antibiotic exposure [88,89,90]. For example, after metronidazole treatment, there have been reports of a decrease in beneficial SCFAs-producing bacteria like *Blautia* spp. and *Faecalibacterium* spp. and an increase in potentially harmful bacteria like *Escherichia* spp. in dogs with CE [21]. The combination of amoxicillin and clavulanic acid also showed a more significant impact on the composition of faecal microbiota compared to amoxicillin alone, showing a decrease in SCFAs-producing *Lactococcus* spp. and *Roseburia* spp. [91]. Dogs receiving amoxicillin, in contrast to those receiving amoxicillin plus ribaxamase experienced changes in their gut microbiota, with increases in Fusobacteria, Firmicutes, and Proteobacteria, and decreases in Actinobacteria and Bacteroidetes [92]. The population of *Enterobacteriaceae*, *Enterococcus* spp., and *Campylobacter* spp. were found to increase, while *Bacteroides* spp. decreased following the administration of amoxicillin [93]. Dogs with CE treated with metronidazole experience changes in the composition of certain bacteria groups, such as Bacteroidetes, Firmicutes, Fusobacteria, and Actinobacteria [88]. Similarly, there were noticeable declines in the abundance of *Bacteroidaceae*, while conflicting findings were obtained for *Clostridiaceae* following tylosin treatment. Following tylosin therapy, certain health-related bacteria, such as *Enterococcus* spp. [57], exhibited an increase, whereas others, including *Faecalibacterium* spp., *Blautia* spp., and *Turicibacter* spp., exhibited a decline [90].

Changes in the faecal metabolome are observed when metronidazole or tylosin is administered [89,90]. In both healthy dogs and dogs with acute diarrhoea, there is an increase in primary bile acids, specifically chenodeoxycholic acid and cholic acid, while secondary bile acids, such as deoxycholic acid and lithocholic acid, decrease [89]. Similarly, in healthy dogs, the administration of tylosin has been shown to elevate the concentration of primary bile acids in faeces [90]. Furthermore, metronidazole treatment has been observed to result in reduced levels of faecal vitamin and antioxidant concentrations, as well as elevated levels of oxidative stress molecules, like ribonic acid and isothreonic acid, in healthy dogs [89].

Interestingly, kittens that had not been exposed to antimicrobials displayed a remarkable resistance to infection with enteropathogenic *Escherichia coli*, while after being infected with enteropathogenic *E. coli* and given a combination of amoxicillin–clavulanic acid and pradofloxacin, all the kittens showed symptoms. However, when they were also given a probiotic containing feline mucosa-associated microbiota, *Enterococcus hirae*, the severity of their gastrointestinal symptoms improved [94]. Cats treated with amoxicillin–clavulanic acid and doxycycline at 2 months of age continued to show a higher abundance of Proteobacteria members and a lower abundance of Firmicutes members up to 4–6 months of age [95]. Interestingly, in cats, reductions in *Enterobacteriaceae*, *Veillonelaceae*, *Prevotellaceae*, and *Porphyromonadaceae* were still observed even after 2 years of discontinuing clindamycin [96], and reductions in cholic acid and deoxycholic acid after one month and two years of the treatment, respectively. In cats, clindamycin had an impact on the levels of various metabolites associated with SCFAs, sphingolipids, amino acid metabolites (like tryptophan and indole-3-lactate), and antioxidant function [96,97]. The administration of amoxicillin–clavulanic acid in adult cats resulted in higher levels of *Enterobacteriaceae* and *Enterococcus* spp. in their faeces while reducing the presence of *Collinsella* spp. and *Bifidobacterium* spp. [98].

### 3.5. Diet

**Figure 3:**
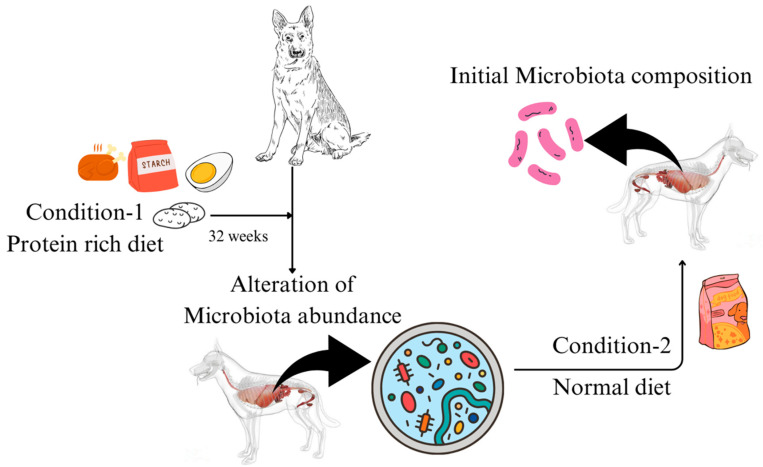
Diet plays a crucial role in the gut microbiome of dogs. An experiment was performed where the dogs were fed the protein-rich diet for 32 weeks, and after that, they were shifted to a regular diet, leading to normalisation of the gut microbiome.

The type and composition of the diet that animal consumes act as a substrate for microbial growth, which leads to the production of SCFAs, secondary bile acids, amino acids, and fat-soluble vitamins released as microbial metabolites [99]. These metabolites, especially SCFAs, exert varied physiological responses in the host, for instance, reducing the inflammation in the intestine [100,101,102,103]. Additionally, the changes in the microbiota in the gut are a result of dietary micronutrients and have become an emerging area of current microbiome research. Molecules like fibre and carbohydrates (Table 3) are fermented by different bacterial species, altering the substrates in the gut and resulting in the growth of specific species and changes in the microbiome and metabolome. Studies conducted by [30], in which for 32 weeks, healthy dogs were fed purified amino acid (protein)-rich diets (Table 4) and digestible starch, which led to an alteration in the species composition, while upon returning to control diet, the microbiome species composition was found to be as to that initial (Figure 3). SCFA producers like *Bacteroides*, *Prevotella*, and *Faecalibacterium* were found to be increased in the weight loss diet (28.1% fibre) fed dogs group. However, the low-fat diet (8.6% fibre) group showed *Faecalibacterium* to be more dominant, suggesting that fibre content impacts the growth of the microorganism. The dietary composition exerts a significant impact on the modulation of gut microbiota growth, as exemplified by the observed correlation between the animal’s feeding regimen and the aforementioned microbial population dynamics.

### 3.6. Probiotics

According to the World Health Organization (WHO), probiotics are described as a live microorganism that, upon administration in prescribed amounts, improves the health of the host [124]. The use of probiotics is generally recognised as valuable for maintaining and promoting gastrointestinal health in both farm animals and companion animals. The popularity of probiotic products for pets underscores the growing interest and awareness among pet owners regarding the potential benefits of probiotic supplementation for their animals’ well-being [62]. Furthermore, its use in companion animals such as dogs and cats has improved the gut microbiota composition, boosting the overall microbial balance to enhance immunologic responses, reduce intestinal inflammation, increase intestinal barrier function, and protect against colonisation by enteropathogens [125].

Bile acids possess detergent properties that can disrupt bacterial cell membranes, cause DNA damage, induce conformational changes in proteins, and chelate calcium and iron [126]. When exposed to bile acids, probiotic bacteria increase the production of certain proteins that help efflux of bile salts or protons. Additionally, they make changes to their overall metabolism in order to counteract the harmful effects of bile acid exposure [127]. Certain types of bacteria, like *Lactobacillus* and *Bifidobacterium*, can resist bile acids, which are linked to the activation of glycolysis [127]. Unconjugated bile acids have stronger antibacterial activity compared to conjugated bile acids. Probiotic bacteria such as *Lactobacillus paracasei* and *Bifidobacterium longum* exhibit greater resistance to bile (taurocholic acid and tauroursodeoxycholic acid exposure) [128]. Conjugated bile acid exposure for a brief period caused the activation of bacterial glycolysis, resulting in an increased growth rate of *Bifidobacterium longum* [128].

**Figure 4:**
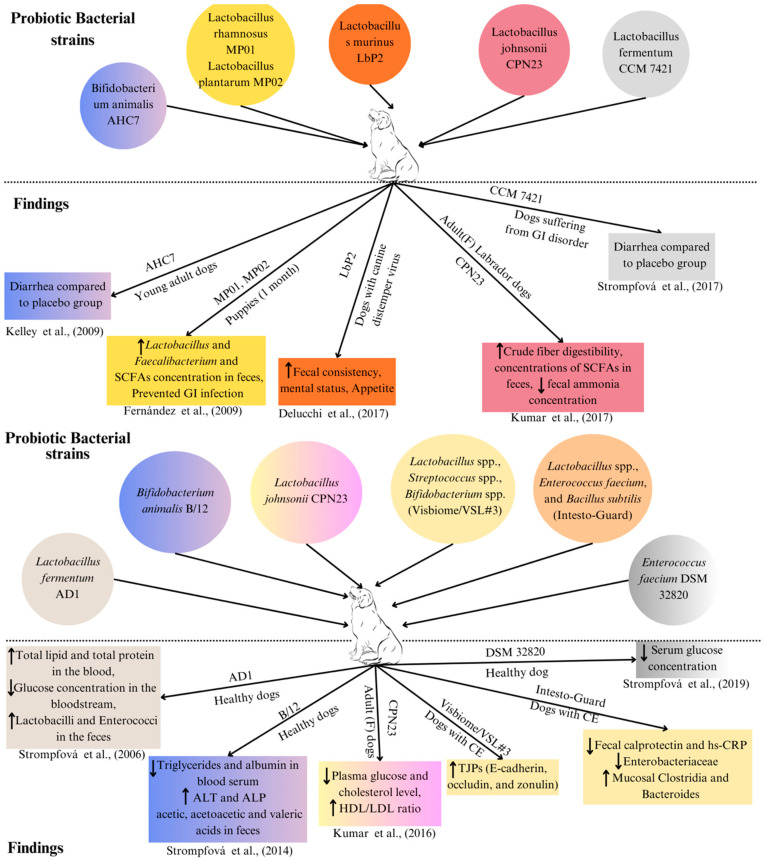
Experiments showing the use of bacterial strains as probiotics and their corresponding finding in the dogs. AHC7: probiotic *Bifidobacterium animalis* strain AHC7 [111,130]; MP01: *Lactobacillus rhamnosus* MP01; MP02: *Lactobacillus plantarum* MP02 [131]; LbP2: probiotic *Lactobacillus murinus* LbP2 [132]; CPN23: *Lactobacillus johnsonii* CPN23 [133]; CCM 7421: probiotic *Lactobacillus fermentum* CCM 7421 [134]; GI: gastrointestinal; HDL: high-density lipoprotein; LDL: low-density lipoprotein; ALT: alanine transaminase; ALP: alkaline phosphatase; AD1: *Lactobacillus fermentum* AD1 [135]; B/12: *Bifidobacterium animalis* B/12 [136]; CE: chronic enteropathy; Visbiome/VSL#3: *Lactobacillus plantarum* DSM 24730, *Streptococcus thermophilus* DSM 24731, *Bifidobacterium breve* DSM 24732, *Lactobacillus paracasei* DSM 24733, *Lactobacillus delbrueckii* subsp. *bulgaricus* DSM 24734, *Lactobacillus acidophilus* DSM 24735, *Bifidobacterium longum* DSM 24736, and *Bifidobacterium infantis* DSM 24737 [54]. TJP: tight junction protein; hs-CRP: high-sensitivity C-reactive protein; DSM 32820: *Enterococcus faecium* DSM 32820 [137]. The upwards arrow (↑) represents “increase” and the downwards arrow (↓) represents “decrease”.

**Figure 5:**
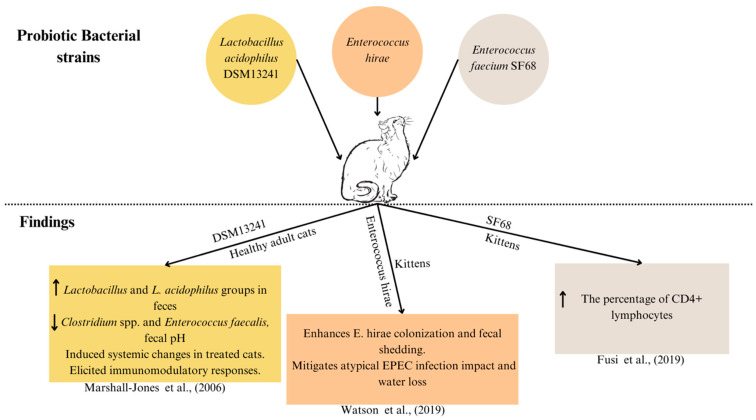
Bacterial strains used as probiotics and their corresponding experimental finding in the cats. Cluster of differentiation 4 (CD4); atypical enteropathogenic *Escherichia coli* (EPEC) [94,138]. DSM13241: *Lactobacillus acidophilus* DSM13241 [139]; SF68: *Enterococcus faecium* SF68 [140]. The upwards arrow (↑) represents “increase” and the downwards arrow (↓) represents “decrease”.

Most probiotics designed for humans and animals contain quantities of *bifidobacteria* and other lactic acid-producing bacteria [129]. A comprehensive list of bacterial strains utilised in both single-strain probiotics and multi-strain mixtures documented in various studies is presented in Table 5 (also in Figure 4 and Figure 5).

**Table 5:** Bacterial strains isolated from the canine and feline with probiotic properties and used for improving gut microbiome.

Bacterial Strains	Amount	Source	Age/Conditions	Assessment	Findings Obtained	Reference
*Bifidobacterium animalis* AHC7	2 × 10^10^ CFU/day	Canine	Young adult dogs having acute diarrhoea	Managing acute diarrhoea	Diarrhoea was reduced compared to placebo group.	[111,130]
*Lactobacillus rhamnosus* MP01, *Lactobacillus plantarum* MP02	10^9^ CFU/day	Canine	Puppies (1 month)	Infection prevention in puppies	Faecal *Lactobacillus* and *Faecalibacterium* were significantly increased. SCFAs in faeces prevented gastrointestinal infection	[131]
*Lactobacillus murinus* LbP2	5 × 10^9^ CFU/day	Canine	Dogs with canine distemper virus (CDV)-associated diarrhoea	Mental and faecal status	Significant improvement in faecal consistency, mental status, and appetite	[132]
*Lactobacillus johnsonii* CPN23	2.3 × 10^8^ CFU/day	Canine	Female Labrador dogs (Adult)	Nutrient digestibility and faecal fermentative metabolites	Crude fibre digestion increased Concentrations of SCFAs in faeces increased Reduction of ammonia in faeces	[133]
*Lactobacillus fermentum* CCM 7421	10^7^–10^9^ CFU/day	Canine	Dogs (having gastrointestinal disorder)	Composition of the faecal microbiome and blood samples	Total protein, cholesterol, and ALT levels in blood samples were increased The population of lactic acid bacteria rose, but the population of clostridia and several Gram-negative bacterial taxa declined. Modification of liquid faeces to regular consistency (dogs with diarrhoea)	[134]
*Lactobacillus fermentum* AD1	3 mL of 10^9^ CFU/mL	Canine	Control healthy dogs	Composition of the faecal microbiome and blood samples	Total lipid and total protein in the blood were significantly increased, while glucose concentration in the bloodstream was notably decreased Faecal *Lactobacilli* and *Enterococci* notably increased	[135]
*Bifidobacterium animalis* B/12	1 mL of 1.04 × 10^9^ CFU/mL	Canine	Control healthy dogs	Composition of the faecal microbiome and Blood samples	Triglycerides and albumin levels in blood serum were significantly lower Increased levels of ALT, ALP, acetic, acetoacetic, and valeric acids in the faeces	[136]
*Lactobacillus johnsonii* CPN23	10^8^ CFU/mL (0.1 mL/kg BW)	Canine	Female dogs (adult)	Assessment of blood sample profile	Decreased plasma glucose and cholesterol level Increased HDL/LDL ratio	[141]
*Enterococcus faecium* DSM 32820	10^9^ CFU/day	Canine	Control healthy dogs	Blood sample profile	Glucose concentration was decreased in serum	[137]
*Lactobacillus acidophilus* DSM 13241	2 × 10^8^ CFU/day	Feline	Healthy adult cats	Improving intestinal health in cats	Lactobacillus and Lactobacillus acidophilus groups were found in higher concentrations in faeces, while *Clostridium* spp. and Enterococcus faecalis were found at lower concentrations Reduced faecal pH and plasma endotoxin concentrations in treated cats, resulting in systemic and immunomodulatory alterations	[139]
*Enterococcus hirae*	2.85–4.28 × 10^8^ CFU/day	Feline	Kittens	Preventing atypical Enteropathogenic *E. coli* (EPEC) in kittens	Promoting intestinal colonisation and faecal shedding of live *E. hirae* upon administration Amelioration of atypical EPEC infection effects on intestinal structure, function, and water loss	[94,138]
*Enterococcus faecium* SF68	5 × 10^9^ CFU/day	Feline	Kittens	*Enterococcus* faecium strain SF68 supplementation on immune function	CD4+ lymphocyte percentage was significantly higher in the treatment group	[140]
*Bacillus subtilis* HH2	5 × 10^9^ CFU/day	Canine	Beagles with orally administered Enterotoxigenic *Escherichia coli* (ETEC)	Intestinal barrier integrity, faecal microbiota, and non-specific immunity	A significant decrease in intestinal barrier marker, diamine oxidase (DAO) levels in plasma Increased serum immunoglobulin IgG, IgM, and IgA levels Increased microbial richness Protection against the ETEC challenge	[142,143]
*Lactobacillus plantarum* DSM 24730, *Lactobacillus paracasei* DSM 24733, *Lactobacillus delbrueckii subsp. bulgaricus* DSM 24734, *Lactobacillus acidophilus* DSM 24735, *Streptococcus thermophilus* DSM 24731, *Bifidobacterium breve* DSM 24732, *Bifidobacterium longum* DSM 24736, and *Bifidobacterium infantis* DSM 24737	*S. thermopilus* 40.55%, Bifidobacteria 12.5%, Lactobacilli 13%, and other excipients 39.05% (112–225 × 10^9^ CFU/10 kg)	Canine	Dogs with CE	Disease activity and mucosal microbiota changes and tight junction protein (TJP) expression	Increased *Lactobacillus* spp. Increased TJP expression (for example E-cadherin, occludin, and zonulin)	[54]
*Bifidobacterium bifidum*, *Enterococcus faecium* and *thermophilus*, and *Lactobacillus acidophilus*, *bulgaricus*, *casei*, and *lantarum*	5 × 10^9^ CFU/day	Feline	Healthy cats	Faecal microbiome and faecal metabolomics	Dysbiosis with alterations in faecal alpha and beta diversity	[97]
*Lactobacillus acidophilus* DSM 32241, *Lactobacillus helveticus* DSM 32242, *Lactobacillus paracasei* DSM 32243, *Lactobacillus plantarum* DSM 32244, *and Lactobacillus brevis* DSM 27961, *Streptococcus thermophilus* DSM 32245, *Bifidobacterium lactis* DSM 32246, *Bifidobacterium lactis* DSM 32247	400 billion cfu of lyophilised bacteria/day	Canine	Healthy dogs	Concentration of faecal immunoglobulin IgA, plasma IgG, and faecal microbiota composition	Decreased abundance of *Clostridium perfringens* Increased abundance of beneficial *Bifidobacterium* and *Lactobacillus* Increased faecal IgA and plasma IgG levels	[144]
*Lactobacillus acidophilus*, *Lactobacillus casei*, *Enterococcus faecium*, and *Bacillus subtilis*	1 billion CFU/mL per 2.2 kg of body weight orally twice daily	Canine	Dogs with confirmed CE	Clinical signs, mucosal microbiota, and inflammatory indices	Decreased levels of inflammatory markers such as faecal calprotectin and high-sensitivity C-reactive protein (hs-CRP) Increased abundance of mucosal Clostridia and *Bacteroides* in colon Decreased abundance of Enterobacteriaceae in colon and ileum	[125]

SCFAs, short-chain fatty acids; CE, chronic enteropathy; TJP: tight junction protein.

### 3.7. Faecal Microbiota Transplantation (FMT)

Faecal microbiota transplantation (FMT) is a recently developed therapeutic method that involves transferring faeces from a healthy donor to a recipient to have the potential to offer a diseased patient with a healthy microbiome. Research has demonstrated that FMT has shown greater effectiveness in managing dysbiosis with promising results than other treatments that modify the microbiome in humans [145] and in dogs with infectious and chronic GI diseases [146]. The faecal microbiota of dogs with diarrhoea treated with FMT as enema and dogs with IBD treated only with corticosteroids show a closer resemblance to the healthy canine faecal microbiota and with a decrease in cholic acid and the percentage of primary bile acids compared to dogs with diarrhoea and IBD treated with metronidazole [21,147]. While dogs suffering from diarrhoea caused by *Clostridium perfringens* toxin A did not respond to antimicrobial treatment, their diarrhoea was successfully resolved when they received FMT through an enema [24]. In a randomised clinical trial, the use of FMT resulted in faster clinical recovery and reduced hospitalisation time for puppies who survived acute hemorrhagic diarrhoea caused by canine parvovirus (CPV) [148]. Also, dogs with CE, when undergoing treatment with a single FMT enema, experienced a notable reduction in their canine IBD activity index (CIBDAI) score following the procedure [149].

## 4. Gut Microbiome and Diseases

### 4.1. Neurological Disorders/Disease

The gut–brain axis, or GBA, is a network of endocrinological, immunological, and neuronal mediators that interacts intricately between the gut and the brain [150]. The gut microbiota, the central nervous system (CNS), and the enteric nervous system (ENS) regulate the GBA [151]. Certain neuroactive substances generated in the gastrointestinal tract can pass through the blood–brain barrier (BBB) and the intestinal mucosal barrier to enter the CNS. These neuroactive molecules bring changes that lead to unfavourable conditions; for example, *Escherichia coli* and *Pseudomonas* can synthesise γ-aminobutyric acid (GABA), an inhibitory neurotransmitter that can cross the BBB. A decline in GABA levels can lead to the occurrence of epileptic seizures, as this inhibitory neurotransmitter (NT) plays a crucial role in regulating neuronal excitability in the mammalian brain. The disruption of GABAergic neurotransmission plays a significant role in the development and manifestation of various neurological disorders, such as epilepsy [152].

**Figure 6:**
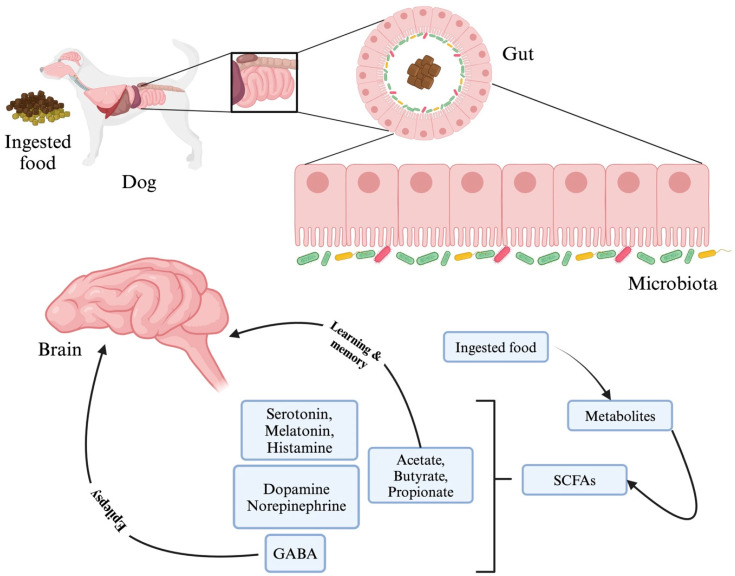
Schematic representation of the conversion of the digested food into metabolites like short-chain fatty acids (SCFAs) and its components like acetate, butyrate, and propionate, which have been shown to indirectly influence the learning and memory process by controlling the energy balance. Decreasing Gamma Amino Butyric Acid (GABA) levels have been shown to generate epileptic seizures. The dopamine–norepinephrine–epinephrine cycle stimulates hormonal and neuronal pathways, while norepinephrine, serotonin, melatonin, and histamine can function as both hormones and neurotransmitters and play a role in excitation/inhibition balance in the brain and epileptogenesis in addition to GABA. This figure was generated using BioRender (www.biorender.com; accessed on 3 May 2024).

As mentioned previously, gut microbes also produce SCFA like propionate, butyrate, and acetate, which act as a source of energy for cell regeneration and mucous production (Figure 6). These SCFAs support intestinal epithelial cell regeneration, mucus formation, and integrity of the BBB. The intestinal fermentation of dietary fibres occurs anaerobically, yielding the highest concentration of these SCFAs. Butyrate serves as colonocytes’ main energy source and reduces inflammation in the intestines to preserve intestinal homeostasis. The generation of SCFAs by microbes is crucial in lowering gut pH and inhibiting potentially harmful bacteria growth. SCFAs have an impact on the CNS by interacting with the free fatty acid receptor (FFAR), a G protein-coupled receptor (GPR) on enteroendocrine cells, including GPR41 (FFAR3), GPR43 (FFAR2), GPR109A/HCAR (hydroxycarboxylic acid receptor)2, and GPR164 [153]. The interaction between SCFAs and these receptors leads to the secretion of various gut hormones and neurotransmitters (NTs), which in turn promotes indirect signaling to the brain via the systemic circulation or vagal pathways. Also, SCFAs play a crucial role in inhibiting the activity of histone deacetylase, which helps to promote the acetylation of lysine residues in nucleosomal histones and increase transcription [153]. Given the potential connection between histone acetylation and inflammation in the CNS, it is plausible that SCFAs may play a role in addressing disruptions to brain immunity.

The hypothesis is that gut microbiota is responsible for Aβ peptide accumulation in intestinal epithelial cells. It was reported by [154] that Aβ peptide and apoB were colocalised in the Golgi apparatus in low-fat and saturated-fat-fed mice. The amyloid β peptide (Aβ) plays a crucial role in initiating the progression of Alzheimer’s disease (AD) through the accumulation and aggregation process. This process can be triggered by either excessive production of Aβ or disruption in its clearance. The Aβ-like peptides are released from the gut microbiome, primarily from the Clostridiales order. Research conducted on a 5xFAD transgenic AD mice model revealed that the administration of prebiotic mannan oligosaccharide resulted in significant improvements that included a reduction in Aβ plaques, cognitive deficits, microglial activation, oxidative stress, and changes in the gut microbiome (GM). Research suggests that alterations in the brain mediated by GM may be influenced by SCFAs, as observed in studies where SCFA supplementation yielded similar results [155]. In another study, the effects of the prebiotic R13 on 5XFAD mice were investigated. The study revealed that this compound, acting as a tropomyosin receptor kinase B (TrkB) agonist, effectively inhibits the proinflammatory C/EBPB/AEP pathway in the gut. Additionally, it was observed that the presence of amyloid-positive signals in the gut was reduced as well [156]. Research has indicated that the KD may have potential benefits in AD [157]. Research conducted on murine models of AD has revealed promising results regarding the potential benefits of the KD, showing to decrease amyloid plaques, enhance memory, and alleviate neurodegeneration and neuroinflammation [158]. The KD can have a positive impact on the composition of gray matter and neurovascular function, boosting the presence of potentially beneficial gut microbiota (*Akkermansia muciniphila* and *Lactobacillus*) while decreasing the levels of potentially pro-inflammatory bacteria (Desulfovibrio and Turicibacter), potentially offering benefits for AD as reported in murine models [159]. A modified Mediterranean–ketogenic diet also exhibited the potential to impact the gut microbiota and SCFA production in individuals with mild cognitive impairment (MCI). Interestingly, these changes were found to be associated with the levels of amyloid measured in the cerebrospinal fluid (CSF) [160].

One of the most prevalent neurological conditions in both people and canines is epilepsy. Whilst studies completed in the UK suggested that 0.62–0.8% of dogs overall have epilepsy, the actual prevalence of the condition is unknown. The presence of gut microbiota (GM)-derived SCFAs can potentially influence the likelihood of experiencing seizures. This influence is believed to occur through the regulation of excitatory/inhibitory neurotransmitters, neuroinflammation, oxidative stress as well as psychosocial stress. When the ketogenic diet (KD) was introduced to epilepsy patients, it was found to have a positive impact on chemical messengers in the brain, such as GABA, agmatine, and monoamines. This led to a decrease in neuronal irritability and ultimately helped to reduce seizures in patients. In the CNS, there is an increase in the production of GABA, while the quantity of aspartate inhibitors decreases [161]. The decrease in aspartate levels caused by ketosis contributes to the activation of glutamate, which is then converted into glutamine. Neuron cells readily absorb glutamine and convert it into GABA, which has an inhibitory effect that helps to decrease oxidative stress [162]. It is worth mentioning that short-term feeding of the KD has also shown positive effects on shifting the metabolome towards a molecular signature that is anti-tumorigenic in dogs, possibly due to decreased abundance of *Erysipelotrichales* and *Lactobacillales* and increased abundance of *Enterobacteriales*, *Fusobacteriales*, *Bifidobacteriales*, *Aeromonadales*, and *Selenomonadales* [163].

Studies on the effects of the KD on seizures in murine models have yielded conflicting results, with some showing no impact, others indicating an increase in seizures, and some suggesting partial protection against seizures [164]. Several factors can influence the varying outcomes of rodent studies, including variations in experimental design, the age of the animals being tested, and the methods used to assess seizure threshold. Additionally, genetic differences between species can also play a role in determining susceptibility to seizures [165]. Several other studies report that KD helps prevent seizures by increasing the presence of important bacterial species such as *Akkermansia muciniphila* and *Parabacteroides merdae* that synergise to reduce the gammaglutamylation of amino acids, enhance the GABA/glutamate ratios in the hippocampus, and ultimately, protect against seizures [166]. As GABA has a role in the antiseizure effects of the KD [164], an altered diet can lead to uneven expression of GABA, which in turn can lead to a seizure.

Intestinal dysbiosis also leads to the severity of epilepsy since immune responses are elevated due to inflammation. Changes in the gut microbiota’s composition have been linked to gastrointestinal disorders in dogs [167], obesity [168], and, more recently, neurological conditions like meningoencephalomyelitis of unknown origin [169], as well as behavioral issues like aggression and phobic disorders [170]. Microbiota function in dogs with epilepsy remains the subject of relatively few investigations. In a study conducted by [171], the faecal samples collected from 13 pairs of dogs, each consisting of a drug-naive epileptic dog and a healthy dog kept on the same diet, were used to assess *Lactobacillus* populations in dogs with idiopathic epilepsy in comparison to healthy dogs. They failed to find any differences between the groups in the relative or absolute abundance of *Lactobacillus* species or large-scale microbial patterns. However, *Firmicutes*, *Bacteroidetes*, *Proteobacteria*, *Fusobacteria*, and *Actinobacteria* were the predominant bacterial populations in both healthy and epileptic dogs. In a study conducted by [172], the companion dogs (n = 29) were studied to understand changes in the composition of microbiota with aging by performing 16s rRNA sequencing from faecal samples, and it was found that higher age was related to lower Fusobacteria, higher cognitive performance was linked to lower Actinobacteria. Reports are stating the role of gut microbiota in the aggressive behavior of dogs, [170] identified *Catenibacterium* and *Megamonas* as major discriminates of aggressive behavior in dogs, later it was also found that *Catenibacterium* and *Megamonasa* are responsible for primary bile acid metabolism and abdominal pains in human [173]. Gut microbiota, thus, has multiple effects on the central nervous via the innervations from the enteral nervous system.

### 4.2. Cardiac Health

**Figure 7:**
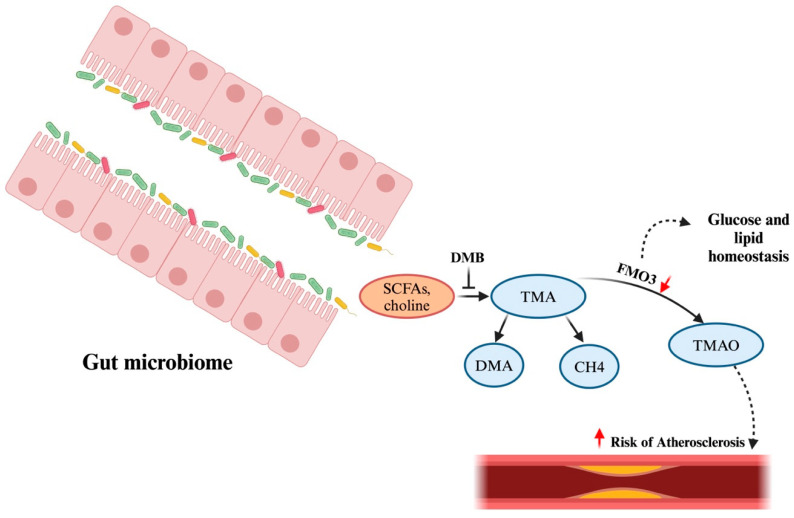
Schematic representation of gut microbiome converting ingested food into metabolites like short-chain fatty acids (SCFAs), choline, and carnitine, which is metabolised into TMA (Trimethylamine), and some part of it is converted into CH4 and DMA + formaldehyde. However, DMB is known to be a repressor of this conversion. TMA is further converted into FMO3 and TMAO after being transported to the liver via the portal vein. Any suppression or deletion in FMO3 leads to altered cholesterol uptake in the intestine and reverse transportation, which might increase the chances of atherosclerosis. This figure was generated using BioRender (www.biorender.com; accessed on 3 May 2024). Trimethylamine-*N*-oxide (TMAO). The upwards red arrow (↑) represents “increase” and the downwards red arrow (↓) represents “decrease”.

Dogs frequently exhibit congestive heart failure (CHF), which is characterised by acute respiratory distress brought on by pulmonary oedema, pleural effusion, or abdominal distension from ascites. Even when their heart disease is well controlled, dogs with congestive heart failure (CHF), especially those with right-sided congestive heart failure (RCHF), are frequently put to death because of severe cachexia and progressive inappetence [174]. A study conducted by Seo et al. [175] demonstrated that dogs with CHF had increased *E. coli* and uncharacterised species of *Enterobacteriaceae*. An increasing amount of research has shown that the gut microbiota and its metabolites play a role in the development and course of cardiovascular disease. In one study by Li et al. (2021), a potential relationship between the gut microbiome and myxomatous mitral valve disease (MMVD) in dogs was observed [176]. It was found that the gut dysbiosis index increases in proportion to the severity of MMVD and is inversely associated with *Clostridium hiranonis*, a key bile acid converter in the gut. Secondary bile acids are significant byproducts resulting from the fermentation of primary bile acids by gut microbes. The main secondary bile acids, deoxycholic acid (DCA) and lithocholic acid (LCA), have the ability to influence the composition of the microbial communities in the gut [177]. The study by Li et al. (2021) also revealed that secondary bile acids may promote the growth of beneficial bacteria but inhibit harmful species. For example, secondary BAs, such as DCA, enhance the growth of *Fusobacterium*, while LCA promotes *Faecalibacterium* growth. In contrast, DCA hinders the growth of *E. coli* [176]. These findings indicate a potential interplay between gut microbiota, gut microbiota-produced metabolites, and the pathophysiological progression of MMVD in dogs [176]. The oral microbiome can influence both these disorders and the composition of the gut microbiota. Even after traditional risk variables have been taken into account, there is still evidence of a favourable association between TMAO and the prediction of cardiovascular risk [178], while SCFAs, on the other hand, help to regulate blood pressure. A significant amount of choline, carnitine, and phosphatidylcholine are broken down into the precursor trimethylamine (TMA) by some gut bacteria (Figure 7). The downstream signaling and metabolism of TMA lead to the production of FMO, which is considered to be a marker for the treatment of cardiovascular disease. In acute myocardial infarction animal models, the gut microbiome, particularly the *Tissierella Soehngenia* genus, the *Synergistetes* phylum, the *Spirochaetes* phylum, the *Lachnospiraceae* family, and the *Syntrophomonadaceae* family, exhibits a greater trend. However, the research on the cardiovascular system is unexplored, and more mechanistic-based studies need to be accounted for to find the risk associated with heart failure.

### 4.3. Chronic Inflammatory Enteropathies in Dogs and Cats

**Figure 8:**
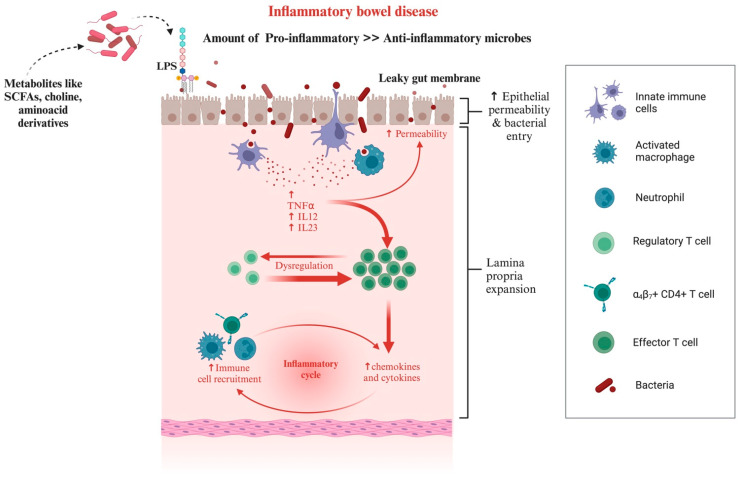
Either ingestion or conversion to LPS or an increase in the ratio of pro-inflammatory to anti-inflammatory microbes can lead to a leaky gut membrane, which is characterised by an elevated response by the immune system. The release of chemokines, cytokines, and interleukins contributes to the progressing havoc leading to inflammatory bowel disease. This figure was generated using BioRender (www.biorender.com; accessed on 3 May 2024).

The intestinal microbiota of dogs and cats is linked to several primary GI disorders associated with dysbiosis. Canine chronic inflammatory enteropathy (CIE) is a common cause of chronic GI signs and histologic inflammation [179]. It is a multifactorial disorder where the interplay between intestinal immunity and environmental factors (diet, microbiota) initiates and drives chronic intestinal inflammation, causing vomiting, diarrhoea, alterations in appetite, and/or weight loss in affected dogs. Different microbiologic studies have shown an increase in intestinal Proteobacteria and a decreased abundance of Clostridiales associated with intestinal inflammation. Using FISH probes, one study discovered that Beagle dogs with persistent diarrhoea had considerably higher *Bacteroides* levels [180]. In a study conducted by Suchodolski et al. [181], wherein the changes in the faecal microbiome were studied, it was revealed that while there was a reduction in *Faecalibacterium* spp. and the phylum *Fusobacteria* during periods of clinically insignificant CIE, there was no significant difference during periods of active disease. In addition to providing the host with nutritional advantages, a balanced intestinal ecology primes and stimulates the immune system and helps defend against invasive intestinal pathogens. In animal model studies involving germ-free animals (rodents), there are morphological and immunological variations between germ-free and conventionally raised animals, demonstrating the effects of the resident microbiota on intestinal structure and function. In the GI tract, residing bacteria can release various compounds, including lipopolysaccharides (LPS), amyloid, and other immunogenic mixtures [125], into the surrounding intestinal environment [38,44,182,183,184]. Immune activation, either by the LPS secretion or an increase in the pro-inflammatory microbes compared to the anti-inflammatory microbes, shapes gut dysbiosis, further contributing to gut inflammation (Figure 8).

The etiology of chronic intestinal inflammation in canines may involve microbial imbalances, potentially serving as both a causative factor and a resultant outcome [38,185]. Dogs afflicted with CE have been observed to manifest a notable reduction in both faecal bacterial richness and diversity [21,102]. Moreover, it has been observed that both luminal and mucosal intestinal bacteria exhibit a shared dysbiotic profile, which is distinguished by a reduction in the abundance of *Clostridium*, *Fusobacterium*, and *Bacteroides*, alongside an elevation in the abundance of Enterobacteriaceae [125,186,187,188] in canine subjects presenting with persistent gastrointestinal symptom. The members of the Enterobacteriaceae family are regarded as pathogenic owing to their capacity to elicit innate immune reactions within the gastrointestinal tract [189]. The comparative analysis of microbial populations in diseased dogs and healthy control (HC) dogs revealed elevated levels of *Bifidobacterium* spp., *Lactobacillus* spp., *Streptococcus* spp., and potentially pathogenic *Escherichia coli* (*E. coli*) in the former group [102].

In another study, dogs subjected to a dietary intervention involving the administration of a supplement comprising a combination of four distinct strains of probiotic bacteria, prebiotics, and IgY exhibited notable alterations in their mucosal microbiota in comparison to dogs who received a hydrolysed diet (placebo) treatment [125]. Dogs that received supplementation exhibited elevated quantities of colonic mucosal *Bacteroides* spp. and *Clostridium* spp. while experiencing a reduction in the abundance of mucosal Enterobacteriaceae compared to dogs administered a placebo [125]. It was observed that there was an overrepresentation of mucosal Enterobacteriaceae in dogs with CE prior to treatment. However, a significant reduction in the abundance of these Enterobacteriaceae was observed in the colonic biopsies of canines that received the supplement [125]. Several other studies have consistently demonstrated a noteworthy decrease in the abundance of *Clostridium* spp. and *Bacteroides* spp. during the initial stages of these conditions [186,187,188]. Research findings indicate that these specific bacterial species have been identified to play a significant and advantageous role in producing SCFAs [40,41,102,190]. The SCFAs, including butyrate, serve as an energy source for colonocytes and play a significant role in upholding the integrity of the intestinal epithelial barrier [191].

### 4.4. Obesity

Pet populations are experiencing a concerning rise in obesity and overweight issues, with many contributing factors at play, such as sedentary lifestyles, high-calorie meals, genetic predispositions, and neutering. The significance of gut microbiota, specifically alterations in bacterial taxa and metabolite profiles, is underscored in relation to obesity [192] and associated diseases in cats and cardiac disease [175], diabetes mellitus [12], and orthopedic diseases [23] in dogs.

Multiple studies have found a reduction in bacterial diversity in the faecal microbiota of obese and overweight dogs compared to dogs with a healthy weight [193]. There was a higher presence of Actinobacteria and *Roseburia* in obese dogs [168]. It was found that the presence of the Actinobacteria class was more pronounced in obese dogs compared to dogs of normal weight. While the gut microbiota of lean dogs was primarily composed of microbes from Firmicutes (85% of the total population), the gut microbiota of obese dogs was mainly composed of bacteria from the phylum Proteobacteria, accounting for 76% of the total [194]. The presence of Gram-negative bacteria could potentially contribute to chronic low-grade inflammation in obese dogs by producing higher levels of intestinal LPS [195]. Overweight dogs, on the other hand, had a greater abundance of the Erysipelotrichi class, mainly due to differences in *Eubacterium* spp. [196]. There was a noticeable decrease in the abundance of Bifidobacteriales and a slight increase in the abundance of Aeromonadales in overweight dogs compared to obese dogs. Overweight dogs showed a higher prevalence of *Prevotella copri* and *Clostridium* compared to obese dogs. Previous studies have found that overweight dogs tend to have a greater presence of *Fusobacteria*, particularly *Fusobacteria perfoetens*, compared to normal dogs [193]. In contrast to overweight dogs, dogs with a healthy weight showed an elevated population of Erysipelotrichales, Erysipelotrichaceae, and Erysipelotrichi while having a reduced presence of Bifidobacteriales. Normal-weight and overweight dogs had a higher representation of the *Blautia*, Lachnospiraceae family, and *Eubacterium biforme* compared to obese dogs. Normal-weight dogs had a higher relative abundance of the Ruminococcus family compared to obese dogs [193,194]. A negatively correlated abundance of *Megamonas* and the weight-loss rate, a noticeable decrease in the presence of Ruminococcaceae, and lower levels of propionic and acetic acid in the faeces of dogs that experienced rapid weight loss, compared to dogs with slower weight loss, demonstrate that obese dogs have faecal bacteria that can produce propionic and acetic acids and may be less responsive to weight loss because they have a greater capacity to extract energy from the diet through SCFAs production [77]. Notably, prebiotics such as fructooligosaccharides have the potential to influence the composition of the gut microbiota positively. An effective treatment approach for obesity-related health problems in companion animals may involve the administration of short-chain fructo-oligosaccharides since it has been associated with enhanced microbial biodiversity and the synthesis of anti-inflammatory butyrate in obese dogs [77,104,168,192,193,194,196,197,198,199,200,201,202].

## 5. Future Prospects and Conclusions

This comprehensive review illuminates the intricate and multifaceted interplay between the gut microbiome and health in companion animals. Despite foundational characterisations, critical knowledge gaps persist regarding longitudinal microbial dynamics, functional host–microbe interactions, microbial biomarkers of health/disease states, standardised methodologies, environmental modulators, and transgenerational inheritance patterns. Addressing these gaps through rigorous research is pivotal for advancing microbiome-based diagnostics, therapeutics, and interventions to optimise health outcomes in companion animal species. Moreover, it is necessary to reevaluate the regular usage of antibiotics in companion animals experiencing GI symptoms, and other approaches that can improve the GI microbiome, such as FMT or advanced probiotics, should be investigated as alternatives. Though several studies evaluated the effects of probiotics like *Bifidobacterium*, *Lactobacillus*, and *Enterococcus* strains in dogs and cats, still more research is needed to understand their effects on the microbiome and their effectiveness in clinical settings.

Thus, in conclusion, gut health dependency can be attributed to parameters starting from microbiome modulation, nutrient uptake and utilisation, immunomodulatory effects, and use of strain-specific probiotics. Collectively, this review has provided a synthesised perspective on the evolving microbiome landscape in companion animals and its pivotal role in holistic health and disease management. Thus, more advanced studies should be envisaged for establishing a concrete link among the microbial species exhibiting synergistic effects in maintaining the health of companion animals.

## Figures and Tables

**Table 1:** Gastrointestinal microbial species of companion animals.

Phylum	Class	Family	Genus/Species
Proteobacteria	Betaproteobacteria	Alcaligenaceae	*Sutterella*
	Gammaproteobacteria	Enterobacteriaceae	*E. coli*
Fusobacteria	Fusobacteriia	Fusobacteriaceae	*Fusobacterium*
Firmicutes	Bacilli	Turicibacteraceae	*Turicibacter*
		Veillonellaceae	*Megamonas*
		Lactobacillaceae	*Lactobacillus*
		Streptococcaceae	*Streptococcus*
	Clostridia	Clostridiaceae	*Clostridium*
		Ruminococcaceae	*Faecalibacterium prausnitzii*
		Lachnospiraceae	*Blautia*
		Peptostreptococcaceae	*Peptostreptococcus*
Bacteroidetes	Bacteroidetes	Prevotellaceae	*Prevotella*
		Bacteroidaceae	*Bacteroides*
Actinobacteria	Coriobacteriia	Coriobacteriaceae	*Collinsella*

Source: Allaway et al. [30].

**Table 2:** Disease and/or symptoms caused by the gut microbiota in companion animals (cats and dogs).

Disease/Disorder	Microbiota Involved	Author
Secretory diarrhea	*Clostridium hiranonis*	[45]
Carbohydrate fermentation	*Bifidobacterium*, *Lactobacillus*, and *Faecalibacterium*	[36]
Prevents leaky gut syndrome	*Clostridiales*	[36]
Protects against excessive inflammation	*Parabacteroides*	[46]
Mitigates CE	*Lactobacillus acidophilus* strains and *Lactobacillus johnsonii* strain	[47]
Intestinal disease (in cats)	*Bifidobacteria* and *Bacteroides* (decrease), *Desulfovibrio* (increase)	[48,49]
Small cell intestinal lymphoma	*Fusobacterium* sp. (increase)	[50]

Chronic enteropathies (CE).

**Table 3:** Effect of fibre and the gut microbiome of domesticated cats and dogs [101].

Impact on Dogs				
Diet Type	Technique	Results	Alterations in Abundance	References
Inulin-type fructans	16S rRNA seq.	Firmicutes, Erysipelotrichaceae, and Turicibacteraceae	Increase	[104]
Beet pulp	16S rRNA seq.	Erysipelotrichi and Fusobacteria	Decrease	[105]
		Firmicutes and Clostridia	Increase	
Yeast cell wall	16S rRNA seq.	*Bifidobacterium*	Increase	[106]
Inulin	16S rRNA seq.	Enterobacteriaceae	Decrease	[106]
		*Megamonas* and *Lactobacillus*	Increase	
Potato fibre	16S rRNA seq.	*Faecalibacterium*, *Lachnospira*, faecal acetate, propionate and butyrate	Increase	[107,108]
		*Prevotella* and *Fusobacterium*	Decrease	
Soybean husk	qPCR	*Clostridium* cluster XI	Decrease	[109]
		Total Lactobacilli, *Faecalibacterium*, *Bacteroides*-*Prevotella*-*Porphyromonas*, and *Clostridium* cluster XIVa	Increase	
**Impact on Cats**				
FOS	qPCR	*Bifidobacterium*	Increase	[110]
	16S rRNA seq.	Actinobacteria	Increase	[111]
GOS	qPCR	*Bifidobacterium*	Increase	[110]
Cellulose	16S rRNA seq.	No changes	—	[111]
FOS and GOS	qPCR	*Bifidobacterium*, total SCFAs, butyrate, and valerate	Increase	[110]
FOS and inulin	16S rRNA seq.	Veillonaceae	Increase	[112]
		Gammaproteobacteria	Decrease	
Inulin	16S rRNA seq.	*Bifidobacterium*	Increase	[113]
		Faecalibacterium and *Fusobacterium*	Decrease	
Pectin	16S rRNA seq.	Firmicutes	Increase	[111]
Wool hydrolysate	16S rRNA seq.	No changes	—	[114]
Mixed insoluble fibres	16S rRNA seq.	Blautia, *Bacteroides*, Turicibacter, acetic and propionic acids	Increase	[115]
		Isobutyric, 2-methylbutyric, and isovaleric acids	Decrease	
Inulin and cellulose	16S rRNA seq.	*Prevotella*, *Bifidobacterium*, Lactobacillus, Megamonas, and unclassified Lachnospiraceae	Increase	[116]
		*Clostridium*, *Fusobacterium*, and *Eubacterium*	Decrease	

FOS, fructooligosaccharides; qPCR, quantitative polymerase chain reaction; SCFAs, short-chain fatty acids; GOS, galactooligosaccharides; rRNA seq., ribosomal RNA sequencing. No changes (—).

**Table 4:** The effect of high protein diets on the species diversity of gut microbiome in companion animals [101].

**Impact on Dogs**					
**Diet Type**	**Results**		**Feed Duration**	**Number of Individuals**	**References**
Bones and raw foods (BARF)	↓ *Bifidobacterium* and *Faecalibacterium*; ↑ Fusobacteria, *Escherichia coli*, *Streptococcus*, and *Clostridium*		4 weeks to 9 years	27	[117]
Red meat	↓ *Faecalibacterium*, *Peptostreptococcus*, *Bacteroides*, and *Prevotella* ↑ *Fusobacterium*, *Lactobacillus*, and *Clostridium*		9 weeks	7	[35]
Raw diet	↑ Richness, evenness, *Clostridium perfringens*, *Clostridium hiranonis*, *Dorea*, and *Fusobacterium varium*		At least 1 year	6	[118]
Kibble with boiled beef	↓ *Faecalibacterium prausnitzii* ↑ *Clostridium hiranonis*, *Dorea*, *Slackia*, and unidentified Clostridiaceae		1 week per combination	11	[119]
**Impact on Cats**					
**Diet Type**	**Results**	**Other Specifications**	**Feed Duration**	**Number of Individuals**	**References**
High-protein low-carbohydrate dry food	↓ *Lactobacillus*, *Bifidobacterium*, and *Escherichia coli*	Kitten, weaning diet	8 weeks	7	[120]
	↓ Actinobacteria, *Bifidobacterium*, *Dialister*, *Acidaminococcus*, *Megasphera*, and *Mitsuokella* ↑ Fusobacteria, *Clostridium*, *Faecalibacterium*, *Ruminococcus*, *Blautia*, and *Eubacterium*	Kitten, weaning diet	8 weeks	7	[116]
	↑ Species diversity; affected 194 metabolic pathways, including amino acid synthesis and metabolism	Kitten, weaning diet	8 weeks	6	[121]
Raw 1 to 3-day-old chicks	↑ *Peptococcus*, *Pseudobutyrivibrio*, and unidentified Lachnospiraceae	Adult	1.3 weeks	5	[122]
Raw	*↑ Clostridium*, *Fusobacterium*, *Eubacterium*, and molar ratio of butyrate	Adult	3 weeks	12	[123]
Raw plus plant fibre	*↓ Clostridium*, *Fusobacterium*, and *Eubacterium*	Adult	3 weeks	12	[123]
	*↑ Prevotella*				
Canned	*↓ Firmicutes*, *Bacteroides*, *Lactobacillus*, and *Streptococcus*	Kitten, weaning diet	9 weeks	10	[67]
	*↑ Fusobacterium*, *Clostridium*, unidentified *Peptostreptococcaceae* and *Prevotellaceae*				
	*↓ Lactobacillus*, *Megasphera*, and *Olsenella*	Adult	5 weeks	16	[67]
	*↑* Species richness, *Fusobacteria*, *Proteobacteria*, *Clostridium*, *Blautia*, *Bacteroides*, and unidentified *Peptostreptococcaceae*				
	↓ *Lactobacillus*, *Bifidobacterium*, and *Collinsella*	Kitten, weaning diet	9 weeks	10	[113]
	↑ *Bacteroides*, *Clostridium*, *Fusobacterium*, genes involved in vitamin biosynthesis, metabolism, and transport	Kitten, weaning diet			

Increase in abundance (↑); decrease in abundance (↓).

## Supplementary Materials

The following supporting information can be downloaded at: https://www.mdpi.com/article/10.3390/microorganisms12091831/s1, Supplementary Figure S1. Worldwide preference for different companion animals (from aquaria population to big mammals). The information was accessed from worldpopulationreview.com (https://worldpopulationreview.com/country-rankings/pet-ownership-statistics-by-country; accessed on 10 August 2024). Supplementary Figure S2. The population of total companion animals and its estimates across the world. The information was accessed from worldpopulationreview.com (https://worldpopulationreview.com/country-rankings/pet-ownership-statistics-by-country; accessed on 10 August 2024). M: Million.
