# Supplementary material for: Decoding the Gut Microbiome in Companion Animals: Impacts and Innovations
Source: Microorganisms. 2024 Sep 4;12(9):1831. doi: 10.3390/microorganisms12091831 (PMC11433972; doi:10.3390/microorganisms12091831)
Supplement: Supplementary file 1 [file microorganisms-12-01831-s001.zip › microorganisms-3132972-supplementary.pdf]

Supplementary Figures

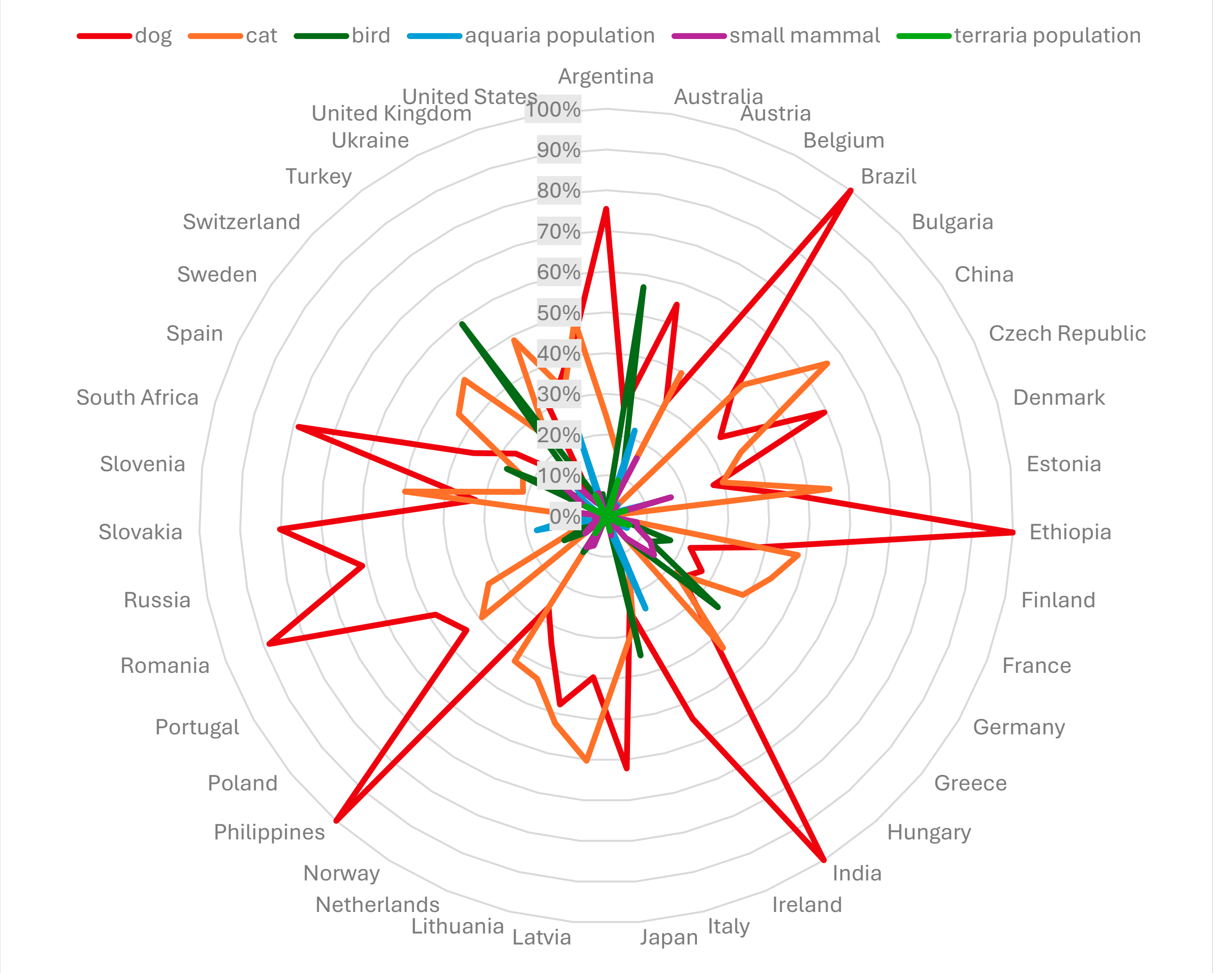

Supplementary Figure S1. Worldwide preference for different companion animals (from aquaria population to big mammals). The information was accessed from worldpopulationreview.com (https://worldpopulationreview.com/country-rankings/pet-ownership-statistics-by-country; accessed on August 10th, 2024).

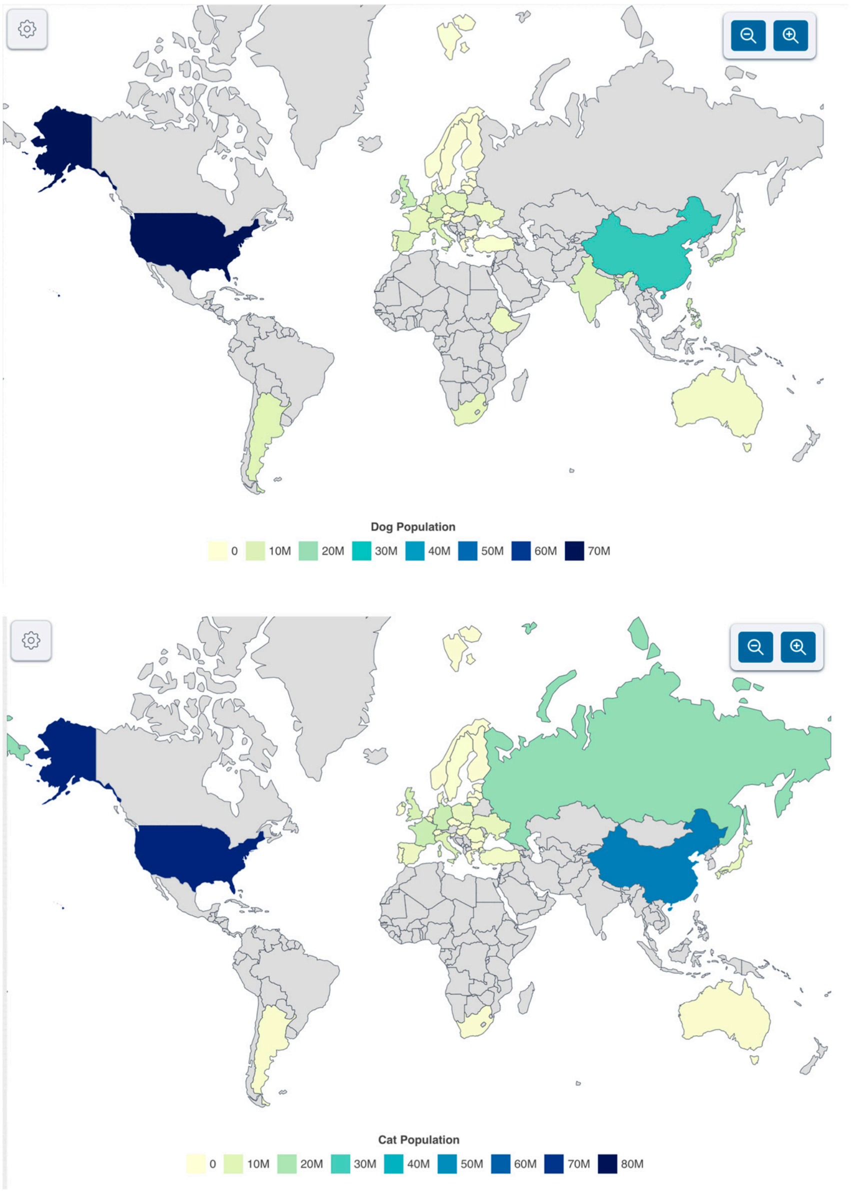

Supplementary Figure S2. The population of total companion animals and its estimates across the world. The information was accessed from worldpopulationreview.com (https://worldpopulationreview.com/country-rankings/pet-ownership-statistics-by-country; accessed on August 10th, 2024). M: Million.
